# In Vivo “MRI Phenotyping” Reveals Changes in Extracellular Matrix Transport and Vascularization That Mediate VEGF-Driven Increase in Breast Cancer Metastasis

**DOI:** 10.1371/journal.pone.0063146

**Published:** 2013-05-01

**Authors:** Arvind P. Pathak, Stephen McNutt, Tariq Shah, Flonne Wildes, Venu Raman, Zaver M. Bhujwalla

**Affiliations:** JHU ICMIC Program, Division of Cancer Imaging Research, Russell H. Morgan Department of Radiology and Radiological Science, The Johns Hopkins University School of Medicine, Baltimore, Maryland, United States of America; University of Chicago, Department of Medicine, United States of America

## Abstract

**Purpose:**

To gain new insights into the relationship between angiogenic factors in breast cancer and their effect on extracellular matrix (ECM) remodeling and metastasis, we characterized and validated the “metastatic signature” of human breast cancer cell lines engineered to overexpress VEGF in terms of *in vivo* MRI-derived angiogenesis and ECM transport parameters.

**Methodology:**

MRI was used to evaluate the effects of overexpressing VEGF-A (VEGF_165_) on tumor angiogenesis and ECM remodeling *in vivo*, for two differentially metastatic human breast cancer cell lines: MCF-7 and MDA-MB-231.

**Principal Findings:**

Overexpression of VEGF elevated vascular volume in both MCF-7-VEGF and MDA-MB-231-VEGF tumors relative to their wild-type counterparts, but vascular permeability was elevated only in MCF-7-VEGF tumors. A significant increase in the volume of extravascular fluid drained as well as the number of ECM drainage voxels was detected in MCF-7-VEGF tumors relative to MCF-7 tumors, but not in MDA-MB-231-VEGF versus MDA-MB-231 tumors. The angiogenic effects of VEGF overexpression in both MCF-7-VEGF and MDA-MB-231-VEGF tumors were validated histologically. MCF-7-VEGF tumors exhibited enhanced invasion and a greater fraction of cancer positive lungs and lymph nodes relative to MCF-7 tumors.

**Conclusions and Significance:**

*In vivo* MRI and histological data demonstrate that VEGF overexpression results in the progression of noninvasive MCF-7 and invasive MDA-MB-321 tumors to a more angiogenic phenotype. However, VEGF overexpression significantly altered ECM integrity only in MCF-7 tumors, causing them to progress to an invasive and metastatic phenotype. This study for the first time demonstrates the concurrent effects of VEGF overexpression and ECM remodeling on metastasis *in vivo*. Collectively, these findings demonstrate that *in vivo* MRI can non-invasively monitor changes in the tumor microenvironment that can potentially predict a cancer’s ability to metastasize.

## Introduction

The tumor microenvironment plays a critical role in several of the phenotypic traits exhibited by breast cancer such as angiogenesis [1], lymphangiogenesis [2], invasion and metastasis [3]. The emerging role of the tumor stroma in the regulation of cancer development [3], [4], new data elucidating the link between interstitial flow and metastasis [5], and the complex micromilieu at the host tissue-tumor interface [6], have warranted the development of imaging approaches to probe their roles noninvasively, and *in vivo* [7].

Of the several known tumor microenvironmental factors, vascular endothelial growth factor (VEGF) has emerged as a cytokine essential for the development of many aggressive phenotypic traits [8]–[10]. VEGF is not only a potent angiogenic factor in breast cancer [11], but its expression level has also been associated with poor prognosis in primary breast cancer [12], [13]. Furthermore, as summarized by Mercurio *et al*., the function of VEGF in breast cancer is not confined to angiogenesis alone, but extends to aiding the progression of breast cancer cells towards a more invasive and metastatic phenotype [14].

While most studies have explored the relationship between angiogenesis and VEGF expression in breast cancer using histologic approaches [11], few have investigated these effects *in vivo* [15], and fewer still have assessed the relationship between VEGF expression, angiogenesis and extracellular matrix (ECM) integrity within the context of metastasis [16]. In one such study using C6-pTET-VEGF165 xenografts in which VEGF overexpression was switchable under tetracycline regulation in rat glioma cells, Dafni et al employed MRI and confocal microscopy to demonstrate that VEGF overexpression elevated vascular permeability, interstitial convection, and lymphatic drain in the hind limbs of nude mice [15]. Here we investigated the effects of VEGF overexpression on angiogenesis and the ECM in an orthotopic model of human breast cancer because the effects of VEGF are strongly modulated by the extant tumor microenvironment [1]. Using an *in vivo* MRI technique that simultaneously assesses angiogenesis and ECM integrity [17], we recently demonstrated that lymph node metastasis in orthotopic breast cancer xenografts was associated with increased regions of extravascular fluid clearance, and an invasive phenotype [7]. Here, we employed this imaging approach in conjunction with multi-parametric histological validation to determine the ability of MRI to “phenotype” VEGF-induced changes in the *in vivo* tumor microenvironment of noninvasive MCF-7 and invasive MDA-MB-231 human breast cancer xenografts engineered to overexpress human VEGF-A. We selected these differentially invasive and metastatic cell lines to determine if VEGF overexpression results in the progression of the noninvasive and weakly metastatic MCF-7 tumors [18], [19] to a more angiogenic and metastatic phenotype as characterized by *in vivo* MRI, histology, and metastatic burden.

## Results

### VEGF Overexpression Makes MCF-7 Cells More Invasive

**Figure 1:**
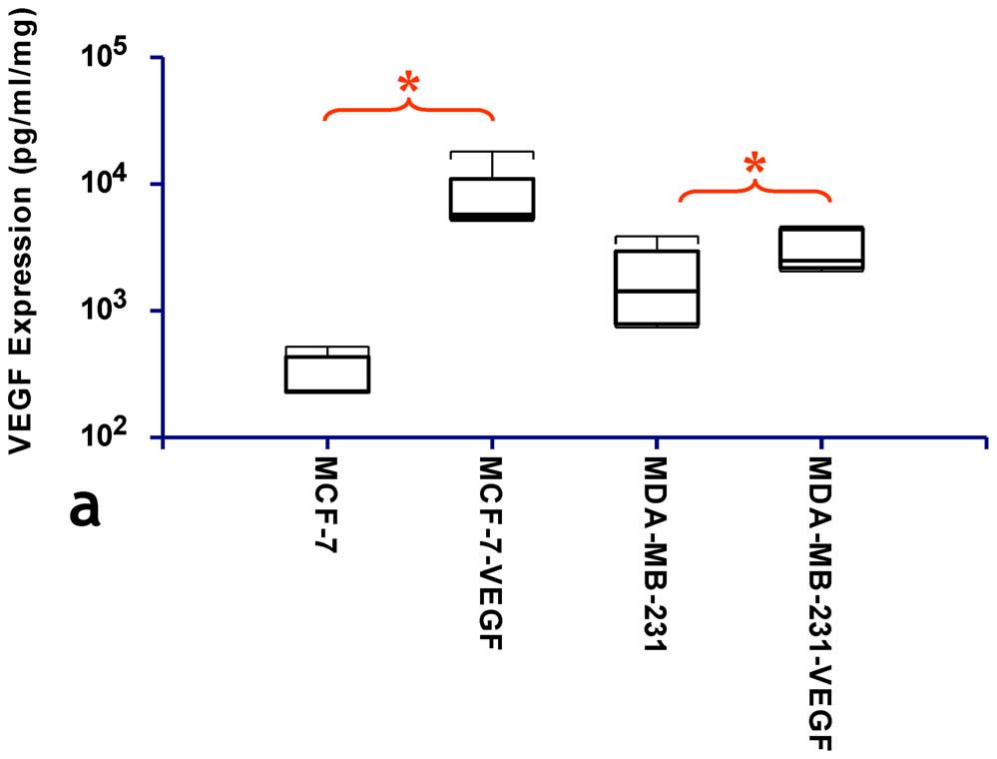
Generation of human breast cancer cell lines overexpressing VEGF. (a) Box-and-whisker plot comparing the VEGF expression levels (expressed on a logarithmic scale) assessed in lysates from MCF-7 and MCF-7-VEGF overexpressing tumors (*p = 0.007 with the one-tailed Mann-Whitney U Test), and from MDA-MB-231 and MDA-MB-231-VEGF overexpressing tumors (*p = 0.0476 with the one-tailed Mann-Whitney U Test), respectively.

**Figure 2:**
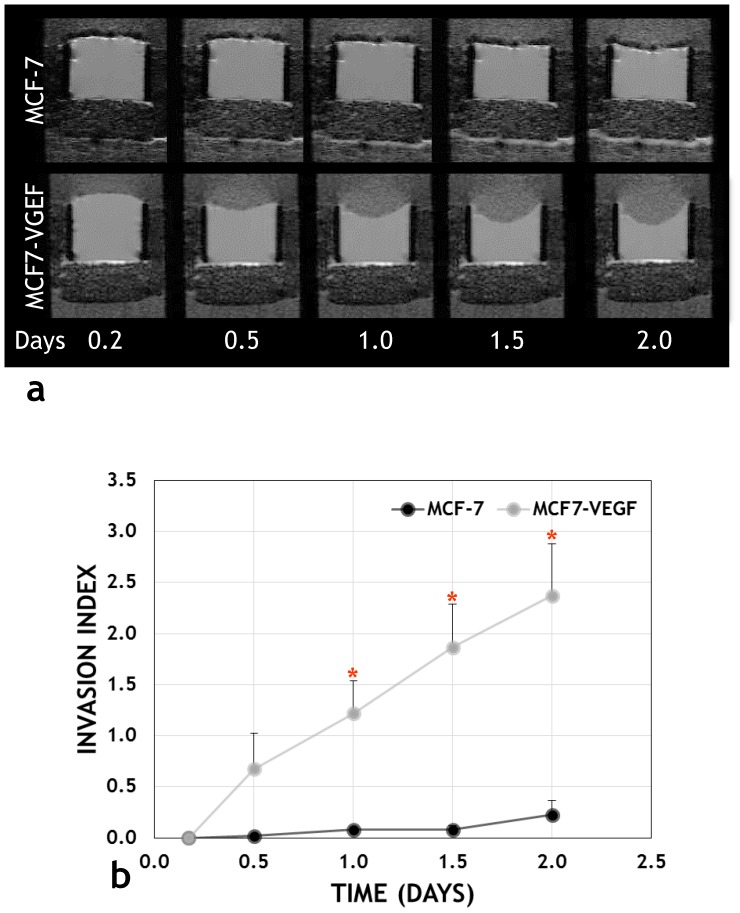
VEGF overexpression increases the invasiveness of MCF-7 cells. (a) Representative MR images of Matrigel® acquired from a MR compatible perfusion chamber over 48 h, which demonstrate significant Matrigel® degradation by MCF-7-VEGF (lower panel) cells in contrast to MCF-7 cells. (b) The time-dependent invasion indices showed a significant (*p = 0.024, p = 0.026 and p = 0.025, respectively with the two-tailed Aspin-Welch Unequal-Variance Test) increase in invasion by MCF-7-VEGF cells compared to MCF-7cells, at 1, 1.5 and 2 days.

Tumor xenografts derived from VEGF overexpressing MCF-7 and MDA-MB-231 cells expressed significantly higher VEGF levels than those derived from the corresponding vector transfected control cells (p = 0.007 and p = 0.0476, respectively) (**Fig. 1a**). **Fig. 2** illustrates the differences in invasion between intact MCF-7 and MCF-7-VEGF cells. MCF-7-VEGF cells exhibited an enhanced ability to invade and degrade matrigel (**Fig. 2a**) and exhibited an elevated invasion index (**Fig. 2b**) at 1, 1.5 and 2 days (p = 0.024, p = 0.026 and p = 0.025 respectively) relative to control MCF-7 cells.

### VEGF Overexpression Induces Robust in vivo Angiogenesis

**Figure 3:**
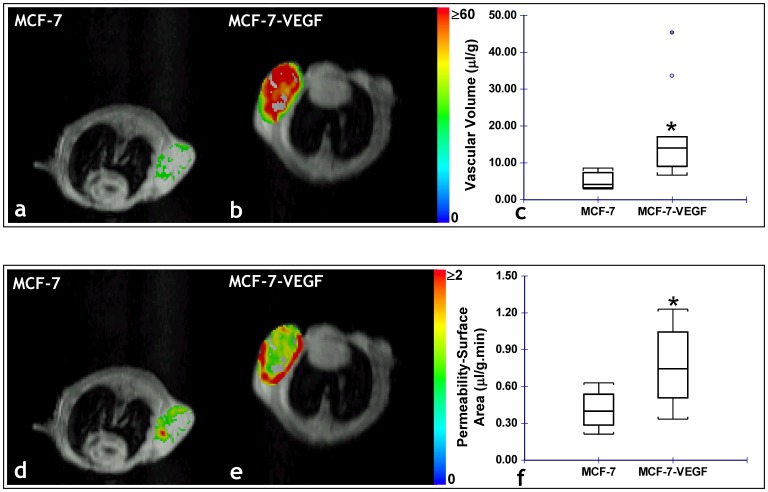
*In vivo* MRI reveals VEGF overexpression alters the angiogenic phenotype of MCF-7 tumors. Representative functional MRI maps of the vascular volume from: (a) MCF-7 and (b) MCF-7 VEGF overexpressing tumor bearing animals. (c) Box-and-whisker plot comparing the vascular volume between MCF-7 (n = 10) and MCF-7-VEGF (n = 12) xenografts. The length of each box is the interquartile range (IQR), while the line through the middle of each box is the median value. The T-shaped lines extending from each end of the box represent the upper adjacent value (i.e. the largest observation ≤ 75th percentile+1.5×IQR) and the lower adjacent value (i.e. the smallest observation ≤ 25th percentile–1.5×IQR), and gray dots denote values outside this range (*p = 0.000059 with the two-tailed Mann-Whitney U Test). Representative functional MRI maps of the permeability-surface area product from: (d) MCF-7 and (e) MCF-7 VEGF overexpressing tumor-bearing animals. (f) Box-and-whisker plot comparing the permeability-surface area product between MCF-7 (n = 10) and MCF-7-VEGF (n = 12) xenografts (*p = 0.0026 with the two-tailed Mann-Whitney U Test).

**Figure 4:**
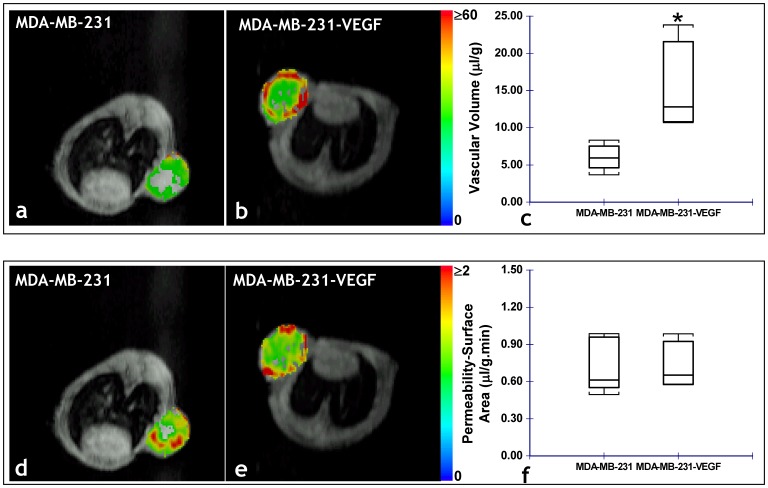
*In vivo* MRI reveals VEGF overexpression alters the angiogenic phenotype of MDA-MB-231 tumors. Representative functional MRI maps of the vascular volume from: (a) MDA-MB-231 and (b) MDA-MB-231 VEGF overexpressing tumor bearing animals. (c) Box-and-whisker plot comparing the vascular volume between MDA-MB-231 (n = 5) and MDA-MB-231-VEGF (n = 4) xenografts (*p = 0.0159 with the two-tailed Mann-Whitney U Test). Representative functional MRI maps of the permeability-surface area product from: (d) MDA-MB-231 and (e) MDA-MB-231 VEGF overexpressing tumor-bearing animals. (f) Box-and-whisker plot comparing the permeability-surface area product between MDA-MB-231 (n = 5) and MDA-MB-231-VEGF (n = 4) xenografts.

**Figure 5:**
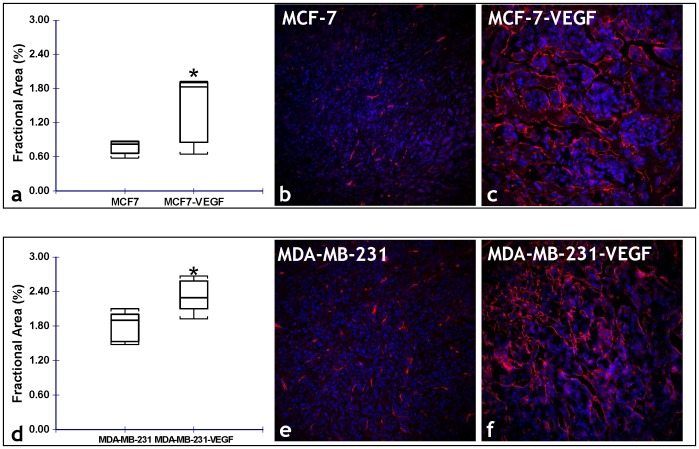
Immunofluorescent validation of VEGF-induced alterations in the angiogenic phenotype. (a) Box-and-whisker plot comparing the stereologically assessed fractional blood vessel area between MCF-7 and MCF-7-VEGF xenografts (*p = 0.0476 with the one-tailed Mann-Whitney U Test). Representative fluorescence (CD34-red, DAPI-blue) photomicrographs (20×) from: (b) MCF-7, and (c) MCF-7-VEGF tumor sections illustrating the difference in the fractional area of CD34 stained blood vessels. (d) Box-and-whisker plot comparing the stereologically assessed fractional vessel area between MDA-MB-231 and MDA-MB-231-VEGF xenografts (*p = 0.0012 with the one-tailed Mann-Whitney U Test). Representative fluorescence (CD34-red, DAPI-blue) photomicrographs (20×) from: (b) MDA-MB-231, and (c) MDA-MB-231-VEGF tumor sections illustrating the difference in the fractional area of CD34 stained blood vessels.

**Fig. 3** illustrates the differences in the angiogenic parameters assessed *in vivo*, between MCF-7 and MCF-7-VEGF tumor xenografts. MCF-7-VEGF tumors exhibited significantly (p = 0.000059) higher vascular volume (**Fig. 3a–c**) and significantly (p = 0.0026) higher permeability-surface area product (**Fig. 3d–f**) than control MCF-7 tumors. In contrast, although MDA-MB-231-VEGF tumors did exhibit a significantly (p = 0.0159) higher vascular volume than the control MDA-MB-231 tumors (**Fig. 4a–c**), there was no significant difference in the permeability-surface area product measured *in vivo* (**Fig. 4d–f**). Consistent with the *in vivo* MR measurements of vascular volume, the stereologically assessed fractional area of CD34*+ve* vessels was significantly (p = 0.0476 and p = 0.0012, respectively) greater in MCF-7-VEGF (**Fig. 5a–c**) and MDA-MB-231-VEGF (**Fig. 5d–e**) tumors relative to the corresponding control tumors. Relatively denser vessel plexuses were apparent in both the MCF-7-VEGF (**Fig. 5c**) and MDA-MB-231-VEGF (**Fig. 5f**) tumors relative to MCF-7 (**Fig. 5b**) and MDA-MB-231 (**Fig. 5e**) control tumors. In contrast to the blood vessel density, there were no significant differences between the stereologically assessed fractional areas of LYVE-1*+ve* lymphatic vessels either between the MCF-7 and MCF-7-VEGF tumors or between the MDA-MB-231 and MDA-MB-231-VEGF tumors.

### VEGF Overexpression Alters the ECM of Non-invasive MCF-7 Tumors

**Figure 6:**
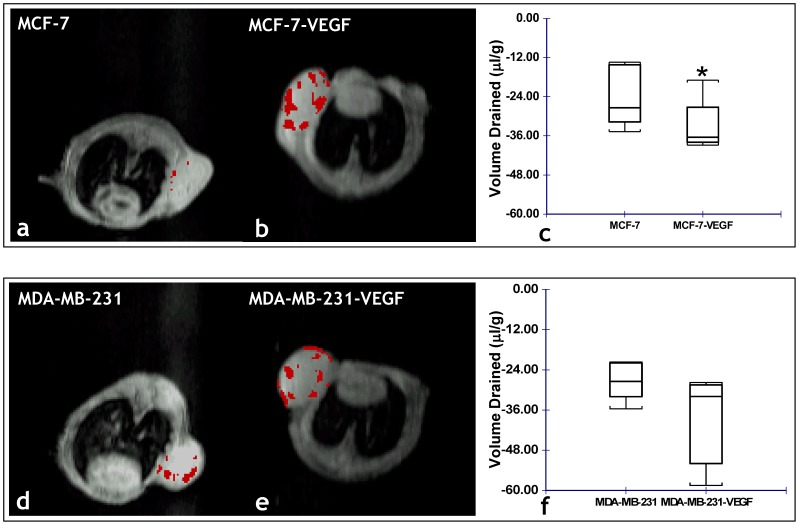
*In vivo* MRI reveals VEGF overexpression alters the ECM of MCF-7 tumors. Representative functional MRI maps of the volume of extravascular fluid drained from: (a) MCF-7 and (b) MCF-7-VEGF overexpressing tumor xenografts. (c) Box-and-whisker plot comparing the volume drained between MCF-7 (n = 5) and MCF-7-VEGF (n = 5) xenografts (*p = 0.0277 with the one-tailed Mann-Whitney U Test). Representative functional MRI maps of the volume of extravascular fluid drained from: (d) MDA-MB-231 and (e) MDA-MB-231-VEGF overexpressing tumor xenografts. (f) Box-and-whisker plot comparing the volume drained between MDA-MB-231 (n = 5) and MDA-MB-23-VEGF (n = 4) xenografts.

Overall, a significantly (p = 0.028) higher percentage of draining voxels and volume of extravascular fluid drained (p = 0.028) were identified for MCF-7-VEGF tumors compared to MCF-7 tumors (**Fig. 6a–c** **).** However, no significant differences were found in the *in vivo* extravascular transport parameters between the MDA-MB-231 and MDA-MB-231-VEGF tumors (**Fig. 6d–f**).

### VEGF Overexpression Transforms the *in vivo* Microenvironment of Non-invasive MCF-7 Tumors to the Metastatic Phenotype

**Figure 7:**
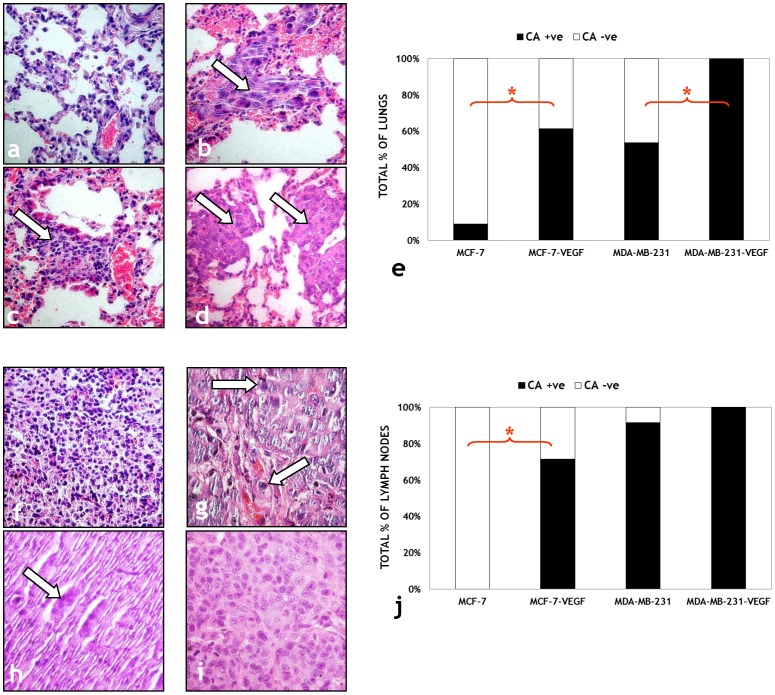
Histological validation of the transformation of non-invasive MCF-7 tumors to the metastatic phenotype following VEGF overexpression. Representative (40×) images of H&E stained lung sections from: (a) MCF-7, (b) MCF-7-VEGF, (c) MDA-MB-231 and (d) MDA-MB-231-VEGF tumor bearing animals. Cancer cells are indicated by solid arrows in each panel. (e) Comparison of the number of animals with cancer positive (CA *+ve*) and cancer negative (CA *-ve*) lungs for each type of breast cancer xenograft. Representative (40×) images of H&E stained lymph node sections from: (f) MCF-7, (g) MCF-7-VEGF, (h) MDA-MB-231 and (i) MDA-MB-231-VEGF tumor bearing animals. Cancer cells are indicated by solid arrows in each panel. (j) Comparison of the number of animals with cancer positive (CA *+ve*) and cancer negative (CA *-ve*) lymph nodes for each type of breast cancer xenograft.

Representative photomicrographs of H&E stained sections obtained from lungs of MCF-7 (**Fig. 7a**), MCF-7-VEGF (**Fig. 7b**), MDA-MB-231 (**Fig. 7c**) and MDA-MB-231-VEGF (**Fig. 7d**) tumor bearing mice demonstrate the differences in lung metastasis observed following VEGF overexpression. There was a significantly (p = 0.013) higher proportion of cancer positive lungs (8/13 vs. 1/11) in MCF-7-VEGF tumor bearing mice compared to MCF-7 tumor bearing mice, and also in MDA-MB-231-VEGF tumor bearing mice compared to MDA-MB-231 tumor (p = 0.019) bearing mice (10/10 vs. 7/13) as summarized in **Fig. 7e**. Representative photomicrographs of H&E stained sections obtained from lymph nodes of MCF-7 (**Fig. 7f**), MCF-7-VEGF (**Fig. 7g**), MDA-MB-231 (**Fig. 7h**) and MDA-MB-231-VEGF (**Fig. 7i**) tumor bearing mice demonstrate that, at the time points examined in this study, there were no significant differences in axillary lymph node metastases of mice bearing MDA-MB-231-VEGF tumors compared to mice bearing MDA-MB-231 tumors (10/10 vs. 11/12) (**Fig. 7j**). However, significant differences were detected in the axillary lymph nodes (5/7 vs. 0/7, p = 0.021) of MCF-7-VEGF tumor bearing animals compared to MCF-7 tumor bearing animals (**Fig. 7j**).

**Figure 8:**
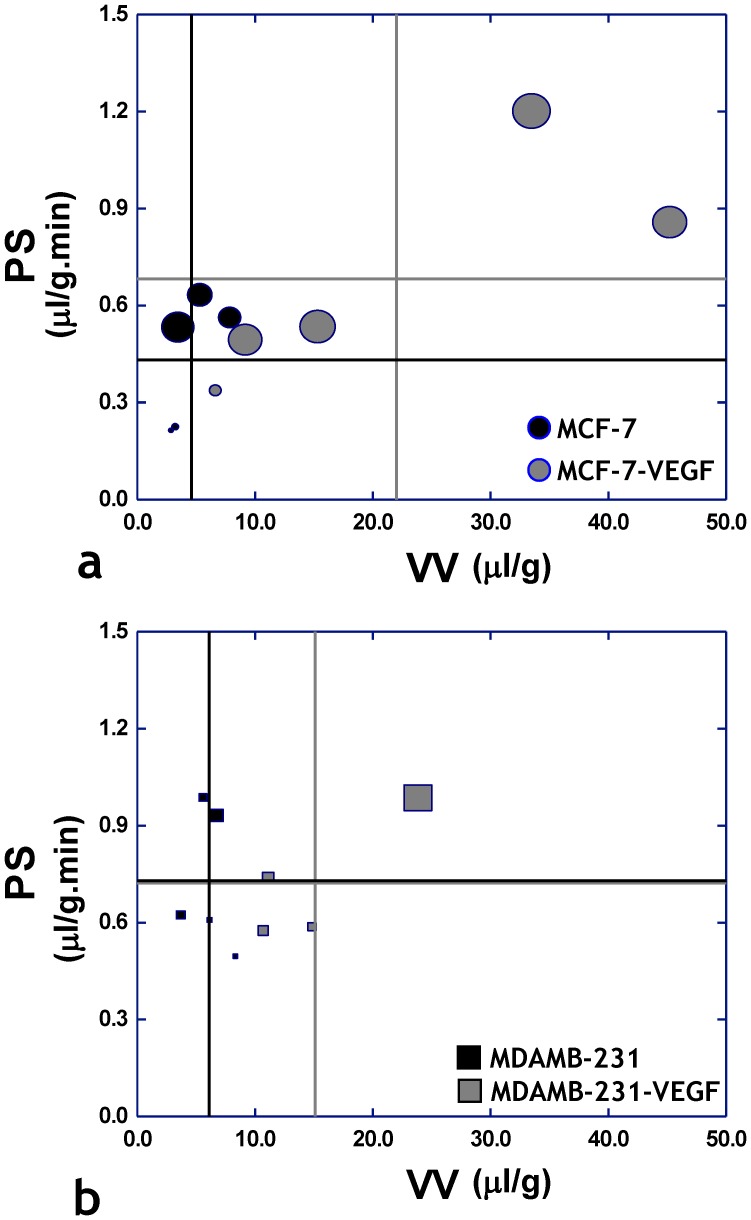
“MRI phenotyping” reveals progression of the non-invasive MCF-7 human breast cancer model to the metastatic phenotype is driven by VEGF overexpression. Scatter plots of permeability-surface area (PS) product *versus* vascular volume (VV) for each tumor type, in which each symbol is scaled according to the volume of extravascular fluid drained. The vertical lines represent the mean value of VV for each tumor type, while the horizontal lines represent the mean value of PS for each tumor type. (a) Scatter plot of parameters measured *in vivo* from MCF-7 (n = 5) and MCF-7-VEGF (n = 5) tumors reveal a trend in the MCF-7-VEGF group towards elevated PS and VV values (gray crosshairs) in conjunction with greater extravascular fluid drainage (larger symbol size) – which collectively indicate the progression of the original MCF-7 phenotype to a metastatic one. In contrast, (b) a scatter plot of parameters measured *in vivo* from MDA-MB-231 (n = 5) and MDA-MB-231-VEGF (n = 4) tumors does not show this trend, but shows the elevation of VV in the MDA-MB-231-VEGF tumors.

**Fig. 8a** summarizes the progression of MCF-7 tumors following VEGF overexpression from a relatively nonaggressive phenotype to an invasive and metastatic one as characterized by changes in both angiogenic and extravascular transport parameters measured *in vivo* using functional MRI. MCF-7-VEGF tumors exhibited higher vascular volume and vascular permeability, as well as elevated ECM drainage (as reflected by the predominantly larger symbol sizes of the overexpressing group) relative to MCF-7 tumors. MDA-MB-231-VEGF tumors exhibited higher vascular volume compared to their MDA-MB-231 counterparts, but there were no appreciable differences in either the vascular permeability or ECM drainage (as reflected by the similar symbol sizes of both groups) between the two groups (**Fig. 8b**).

## Discussion

Tumors derived from stably transfected MCF7-VEGF and MDA-MB-231-VEGF cells expressed elevated levels of VEGF compared to their empty vector transfected counterparts. Consistent with VEGF’s role as a powerful angiogenic factor [10], the *VV* measured *in vivo* for both MCF7-VEGF and MDA-MB-231-VEGF tumors was significantly higher than that of the respective wild-type tumors, an observation corroborated by the stereological measurements of fractional vessel area and other investigators [20]. The potent effect of VEGF on vascular permeability [10], [21] was clearly evident from the elevated *PS* measured *in vivo* for the MCF7-VEGF tumors relative to wild-type MCF-7 tumors. However, VEGF overexpression did not significantly affect *PS* in MDA-MB-231-VEGF tumors relative to wild type MDA-MB-231 tumors. This is consistent with the higher endogenous VEGF levels known to exist in MDA-MD-231 xenografts relative to MCF-7 tumors [22], and with studies demonstrating that VEGF-induced vascular permeability is modulated in a dose-dependent manner and varies between tumor types [23]. While the effect of water exchange on the dynamic contrast enhanced (DCE) MRI parameters is well known [24], the model in this study assesses albumin-(GdDTPA) kinetics within the extravascular space of the tumor. Our results are consistent with observations of increased fractional blood volume and PS in MCF7-VEGF tumors measured using water exchange insensitive DCE computed tomography [25].

The upregulation of key matrix metalloproteinases (MMPs) confers a highly invasive phenotype on wild type MDA-MB-231 tumors [26] and can also explain why the number of draining voxels and *EV* measured in MDA-MB-231-VEGF tumors was not significantly different from that of MDA-MB-231 tumors. Since MDA-MB-231-VEGF tumors derived from cells engineered to overexpress VEGF exhibited a modest increase of VEGF (less than two-fold compared to the 20-fold increase in MCF-7-VEGF tumors), this may also explain the absence of a significant increase in permeability or macromolecular transport. In contrast, MCF-7-VEGF tumors exhibited a larger number of draining voxels, as well as a greater *EV* than that of wild-type MCF-7 tumors indicating a loss of ECM integrity with VEGF overexpression. These data are consistent with recent reports of the roles of VEGF as a potent activator of fibrin degradation [27] and extracellular proteolysis [28]. We found that overexpressing VEGF significantly increased the ability of intact cancer cells to invade and degrade ECM in a cell-perfusion system. These observations are also consistent with the extracellular regulation of VEGF by MMPs during ECM remodeling [29] and initiation of the “angiogenic switch” [30]. While size-matched tumors were employed in each imaging group, the impact of tumor growth rate differences on the ECM independent of the effects of VEGF cannot be ruled out. Since the bioavailability of matrix-bound VEGF is regulated extracellularly by MMPs that in turn are modulated by various microenvironmental factors including growth cues [29], it is extremely challenging to deconvolve these effects using *in vivo* imaging methods.

Interestingly, we did not observe any significant difference in lymphangiogenesis either between MDA-MB-231 and MDA-MB-231-VEGF tumors or between MCF-7 and MCF-7-VEGF tumors. This absence of VEGF-A induced lymphangiogenesis was observed by Cao *et al*. in a mouse corneal assay [31] and is consistent with results from studies in primary breast tumor samples from patients in which lymphangiogenesis was primarily induced by VEGF-D [32]. In contrast, Nagy *et al*. [8] and Hirakawa *et al*. [33] both demonstrated induction of lymphangiogenesis in response to VEGF-A overexpression, and a study of archived primary invasive breast cancer samples demonstrated a high correlation between VEGF-A expression levels and lymphatic vessel density [34]. There are two possible explanations for these differences. First, Nagy *et al*. injected an adenovirus engineered to express VEGF-A into the mouse ear, while Hirakawa *et al*. employed a chemically induced skin cancer model in transgenic mice that overexpressed VEGF-A in the skin. Both of these are distinct microenvironments for lymphangiogenesis compared to the mammary fat pad used in this study. The large amount of extant data demonstrating that breast tumors inoculated into different tissue beds exhibit vastly different angiogenic responses [1], combined with the profound effect of the tumor microenvironment on both lymphangiogenesis [35] and angiogenesis [4] prompted us to employ orthotopic breast cancer models in this study. Second, since tumors were excised in their entirety with little connective tissue, it was not possible to distinguish between intratumoral (ITL) and peritumoral lymphatics (PTL), and all LYVE-1*+ve* (CD34*–ve*) vessels were included in the analysis.

Our data are consistent with recent findings by Shield *et al*., demonstrating that interstitial flow greatly increased tumor cell migration in a chemokine receptor dependent but lymphatic independent manner [5]. In that study, tumor cells employed interstitial flow to modulate chemokine gradients to chemotract towards draining lymphatic vessels, despite being too far to sense chemotactic signals from the lymphatics. This may explain why we observed a significant increase in lymph node metastases for MCF-7-VEGF tumors without a concomitant increase in lymphangiogenesis or MMP production. The fact that we could not differentiate ITL from PTL may also account for this discrepancy, although studies characterizing tumor ITL and PTL in animal models and patient samples have found both, positive and negative correlations of lymphangiogenesis with lymph node metastasis [36].

A significant finding was that MCF-7-VEGF tumors, with their newly acquired “angiogenic phenotype” in conjunction with their “remodeled” ECM exhibited a *de novo* threat of metastatic dissemination. MCF-7-VEGF tumors exhibited significantly higher lung and lymph node metastases relative to their non-invasive, weakly metastatic wild-type counterparts. For MCF-7-VEGF tumors, the elevated vascular permeability combined with increased interstitial fluid drain, conferred a metastatic phenotype resembling the MDA-MB-231 tumors. These findings are consistent with recent reports demonstrating the enhancement of tumor cell invasion and migration by fluid flow in the tumor [37] and mechanical stress-induced changes in the tumor microenvironment [38].

MDA-MB-231-VEGF tumors showed increased metastasis to the lung, whereas the incidence of lymph node metastasis was already high in MDA-MB-231 tumors and increased from 95% to 100%. These data suggest that the increased vascular volume observed in MDA-MB-231-VEGF tumors played an important role in hematogenous metastasis to the lungs, since neither permeability, number of draining voxels or *EV* increased significantly with VEGF overexpression in MDA-MB-231 tumors.

Prior *in vivo* MRI studies of VEGF overexpressing cancer cells have been conducted in in C6-pTET-VEGF165 xenografts inoculated in the mouse hind limb [15], or have demonstrated that the acute response of the tumor vasculature to intradermally administered VEGF_165_ was vasodilation, hyperpermeability and lymphatic uptake [39]. Since Monsky *et al* unequivocally demonstrated that the same breast tumor cell line implanted either into the mammary fat pad or the brain exhibits diverse angiogenic responses [40], in this study we investigated the effects of VEGF overexpression in an orthotopic model of human breast cancer. Due to this dependence of the effects of VEGF on the tumor micromilieu, the findings from our study have important implications for the design of novel anti-metastatic and antiangiogenic therapies designed to modulate the native tumor microenvironment.

Finally, recently discovered non-angiogenic functions of VEGF in breast cancer [14] may help explain the “metastatic” phenotype of MCF-7-VEGF tumors. Specifically, increased VEGF production by tumor cells has been suggested to act in an autocrine manner to directly promote epithelial cell survival [41]. Investigators have also demonstrated that VEGF influences breast cancer invasion and migration via two mechanisms *in vitro*. First, it can promote invasion in an autocrine manner by regulating the expression of chemokine receptors that enable tumor cells to migrate along chemokine gradients [42], [43], and second it can contribute to tumor progression by inhibiting the activity of endogenous suppressors of migration [44]. *In vivo* measurements of these phenomena are crucial to understanding this interplay, as our data clearly demonstrate that MCF-7-VEGF cells were more invasive and MCF-7-VEGF tumors were more metastatic than control MCF-7 cells and tumors.

Analogous to the gene expression profile employed by Brown *et al*. to characterize the phenotype of the vascular stroma in different kinds of breast cancer [3], and the “tripartite arrangement of an invasive carcinoma cell, a macrophage, and an endothelial cell” proposed by Robinson *et al*. of [45] as a potential prognostic marker of metastasis in human breast cancer, here we were able to characterize the “metastatic signature” of transgenic human breast cancer cell lines engineered to overexpress VEGF-A using *in vivo* MRI parameters. While these findings have significant potential for cancer prognosis, it should be borne in mind that the lack of FDA approved macromolecular MR contrast agents currently limits the direct clinical translation of this imaging approach. Other hurdles to translation include the long imaging times necessary to track the kinetics of the contrast agent in the ECM and the need for invasive endpoints (e.g. biopsy samples) to validate the utility of the vascular and extravascular parameters as *in vivo* clinical biomarkers.

In summary, our findings demonstrate that *in vivo* MRI can non-invasively monitor changes in the tumor microenvironment, classify its phenotype and potentially predict a cancer’s readiness to metastasize.

## Methods

### Ethics Statement

All animals were handled in accordance with good animal practice as defined by the relevant national and/or local animal welfare bodies, and all animal work was conducted under a protocol approved by the Institutional Animal Care and Use Committee (IACUC) of Johns Hopkins University. The Johns Hopkins University animal facility is accredited by the American Association for the Accreditation of Laboratory Animal Care and meets National Institute of Health standards as set forth in the “Guide for the Care and Use of Laboratory Animals” (DHHS Publication No. (NIH) 85–23, Revised 1985).

### Tumor Cell Line Generation and Characterization

cDNA for VEGF_165_ (pHUVEGF.21) from Genentech Inc. (South San Francisco, CA) was cloned into the eukaryotic expression vector pCR3.1 under control of a constitutive CMV promoter. MCF-7 and MDA-MB-231 human breast cancer cells obtained from ATCC (Manassas, VA) were stably transfected with either VEGF_165_ or empty-vector construct, and a stable clone selected. Expression of VEGF_165_ in orthotopic tumor xenografts derived from these cell lines was routinely checked, and whole tumor protein quantified, using a colorimetric DC protein assay (Bio-Rad Laboratories, CA) of tumor lysates.

### Detection of VEGF-induced Tumor Cell Invasion

We employed a MRI-compatible cell-perfusion assay to quantitatively assess VEGF-induced invasion in the MCF-7 cell line. A detailed description of the MR cell perfusion system can be found in Ackerstaff et al [46]. Briefly, four days prior to the MR experiments cells were seeded on Biosilon (Nunc, Denmark) beads at a cell density of 1.5×10^6^ cells per 0.5 ml of beads in Petri dishes (Nunc, Denmark) and grown to approximately 60% confluence. A chamber containing Matrigel® (Sigma-Aldrich, St. Louis, MO) at a concentration of 8.8 mg/ml, which was part of the MR-compatible cell perfusion assay, was used to determine the degradation and invasion of ECM by the cancer cells. Two layers of perfluorocarbon doped alginate beads were interspersed within the layers of cancer cells on Biosilon beads, to monitor the oxygen tension in the sample using ^19^F MR relaxometry. The following series of MR experiments were performed on a 9.4 T MR spectrometer (Bruker, Billerica, MA) every 12 h and up to 48 h. Proton MRI was performed to evaluate the overall sample preparation, to visualize the geometry of the ECM gel, and to detect changes in the integrity of the ECM gel due to invasion and degradation by cancer cells. One-dimensional (1D) 1H MR profiles of intracellular water, acquired along the length z-axis of the sample by diffusion weighted 1D ^1^H MR imaging. These profiles were used to derive an invasion index, by quantifying the number of cells invading into the ECM. The invasion index *I(t)* at time ‘*t*’ was calculated according to: I(t) = I_p,7 mm_(t)/I_p_(t) - I_p,7 mm_(t_0_)/I_p_(t_0_).

Where *I_p,7 mm_(t)* and *I_p,7 mm_(t_0_)* is the integral value of the signal at time *t* and *t_0_* respectively, obtained by integrating the intracellular water signal over a 7 mm region starting at the base of the ECM chamber, and *I_p_(t)* and *I_p_(t_0_)* is the integral of the profile of the entire sample at time *t* and *t_0_*, respectively. Here, *t_0_* refers to the first imaging time point which is ∼2 h after the sample has been loaded into the spectrometer. The oxygen tension was measured from slice-selective 1D ^19^F inversion recovery MR experiments as described in [46].

### Tumor Model and Inoculations

VEGF overexpressing MCF-7 tumors (MCF-7-VEGF) inoculated in the upper left thoracic mammary fat pad of 12 female severe combined immune deficient (SCID) mice, and wild type MCF-7 tumors inoculated in 10 SCID mice, were investigated. VEGF overexpressing MDA-MB-231 cells (MDA-MB-231-VEGF) inoculated in five SCID mice, and wild type MDA-MB-231 cells were inoculated in five SCID mice, were studied. Tumor cells were inoculated in 0.05 ml Hanks balanced solution (HBSS, Sigma, and St. Louis, MO) at 10^6^ cells/0.05 ml. Since growth of MCF-7 cells is estrogen dependent, a 17B-estradiol pellet (0.18 mg/pellet, 60 day release, Innovative Research of America, Sarasota, FL) was inserted in the right flank using a trocar.

### General in vivo MRI Protocol

Tumors were imaged *in vivo* 5–6 weeks post-inoculation, on a 4.7 T Bruker instrument using a RF coil around the animal’s torso. The tail vein of each animal was cannulated for i.v. administration of the macromolecular contrast agent albumin-(GdDTPA) (gadolinium diethylenetriamine pentaacetic acid, molecular weight ∼90,000) that was synthesized in our laboratory based on the procedure originally described by Ogan *et al* [47]. Multi-slice relaxation rate maps were obtained using a saturation recovery method combined with fast-T_1_ SNAPSHOT FLASH imaging (flip angle = 10° and TE = 2 ms), as described in [22]. Five to eight (1 mm) coronal slices of the mouse cross-section including the tumor were imaged with an in-plane resolution of 250×250 µm^2^, 32 mm field of view, 8 averages, and four relaxation delays (100, 500, 1000 and 7000 ms). Images were acquired in two phases: an “early phase” comprised of images obtained before administration of 0.2 ml of 60 mg/ml albumin-(Gd-DTPA) in saline and repeated every 7 min, starting at 3 min post-injection, lasting up to 31 min. Since clearance of macromolecules in tumors by lymphatics or convection is a prolonged process [15], [48], [49], data were continuously acquired for up to 140 min post-contrast in a second block. This block of images corresponded to the “late-phase” of albumin-(GdDTPA) kinetics, as it consists of slower extravascular drainage events within the tumor ECM. Following MRI, mice were sacrificed and blood T_1_’s determined from samples taken from the inferior vena cava. Finally, tumors, lungs, ipsilateral and contralateral axillary lymph nodes were excised and fixed in formalin.

#### Detection of Vascular and Extravascular Transport Parameters

In both, “early-” and “late-phases” of the MR experiment, albumin-(GdDTPA) uptake was modeled as a linear function of time and analyzed using a biphasic image analysis technique as described in [17]. This analysis classifies voxels as being either “pooling” (i.e. voxels in which albumin-(GdDTPA) accumulates over time), or “draining” (i.e. voxels from which albumin-(GdDTPA) is cleared over time). Four *in vivo* parameters were computed for each voxel in the tumor: *VV* (vascular volume) and *PS* (permeability surface area product) determined from the “early-phase”, while the *FR* (flux rate) and *EV* (apparent exudate volume) determined from the “late-phase”. A negative *EV* represents drainage of the albumin-(GdDTPA), while a positive *EV* represents albumin-(GdDTPA) pooling [17]. Images were analyzed using the Analysis of Functional Neuro-Images (AFNI) program on a Linux workstation [50]. The median value of each MR parameter was determined for every tumor in each group of animals. A two-tailed, non-parametric Mann-Whitney U test was employed (α = 0.05) to determine if median vascular/extravascular MRI parameters for VEGF overexpressing tumor-bearing animals were significantly different from those for wild-type tumor bearing animals. Sample sizes for statistical comparisons of *in vivo* data are indicated in corresponding figure legends. Since determination of the late-phase of contrast agent kinetics requires ∼3 h of imaging and continuous anesthesia, five animals each from the MCF-7-VEGF (n = 12) and MCF-7 (n = 10) groups underwent the ‘late-phase’ MRI protocol for determining extravascular transport parameters. Finally, one MDA-MB-231-VEGF tumor was excluded from analysis of extravascular transport parameters because it exhibited motion artifact during *in vivo* MRI.

### Immunofluorescence Microscopy Protocol

Adjacent 10 µm frozen tumor sections were cut onto glass slides and lymphatic vessels were immunostained using an antibody to the lymphatic endothelial hyaluronan receptor LYVE-1 (Research Diagnostics Inc., Flanders, NJ) [51]. Blood vessel endothelia were detected on the same tissue with anti-mouse CD34 (clone MEC14.7) antibody (Cell Sciences, Canton, MA). Slides were counterstained with DAPI (Molecular Probes Inc., Eugene, OR) and cover-slipped. Slides were imaged on a Nikon ECLIPSE-TS100 microscope (Nikon Instruments Inc., NY) with the appropriate filters for detecting immunofluorescence. Regions-of-interest (ROIs) were digitized at 20× using a SPOT INSGHT™ CCD camera (Diagnostic Instruments Inc., MI).

#### Morphometric Image Analysis of Tissue Sections

Automated morphometric analysis of each digitized fluorescent image was performed using a macro written by us for ImageJ® (v1.37, Rasband, W.S., U. S. National Institutes of Health, Bethesda, Maryland, USA, http://imagej.nih.gov/ij/, 1997–2011). Briefly, grayscale images were normalized, smoothed, binarized and morphometrically closed. Binarized objects (excluding objects at the image edges) >100 pixels were counted as features, i.e. LYVE-1*+ve* or CD34*+ve* structures. Multiple images corresponding to non-overlapping lymphatic and blood vessel ROIs for each tumor were analyzed and the fractional area occupied by either LYVE-1*+ve* or CD34*+ve* structures stereologically computed [52]. A non-parametric one-tailed Mann-Whitney U test for independence of medians was used to determine if these parameters were significantly (α = 0.05) elevated in VEGF over-expressing than in wild-type tumors.

#### Assay for lymph node and lung metastasis

Tumor positive lymph nodes and lungs from all animals were scored by microscopic examination of H&E stained sections, and significant differences (α = 0.05) between the cancer cell *+ve* fractions for wild-type and VEGF over-expressing tumors evaluated using a two-tailed Fisher’s Exact test.
